# Working memory capacity in naturally cycling women and oral contraceptive users

**DOI:** 10.1192/j.eurpsy.2024.430

**Published:** 2024-08-27

**Authors:** M. Kowalczyk, M. Kornacka, I. Krejtz

**Affiliations:** ^1^Psychology, SWPS University, Warsaw; ^2^Psychology, SWPS University, Katowice, Poland

## Abstract

**Introduction:**

Miyake and Friedman’s model (2012) presents three core executive functions: inhibition, updating and shifting. Updating refers to working memory (WM) because it involves passive storage but also active manipulation of information. According to a review by Hampson (2018), the literature is consistent on the fact that 17b-estradiol (the most prevalent type of estrogen in women of reproductive age) is associated with improved WM. Levels of estradiol are at their highest in the follicular phase of the menstrual cycle. The use of OC is linked with a noticeable decrease in levels of estradiol and progesterone (Hampson, 2020). Nevertheless, combined OC contain synthetic steroids, usually ethinylestradiol and progestins which can be androgenic or anti-androgenic. Androgenic progestins are derived from testosterone while anti-androgenic progestins block androgen receptors (Raudrant & Rabe, 2003). The review by Beltz (2022) concluded that the use of androgenic OC is linked to an enhanced performance in spatial WM whereas anti-androgenic OC are linked with an impaired performance. When it comes to verbal WM, OC use is related to an enhancement in performance, irrespective of androgenicity.

**Objectives:**

To measure the differences in WM capacity between NC women and OC users at two times points in one menstrual cycle (follicular and luteal phases for NC women).

**Methods:**

78 women (18-45; *M*= 28.93, *SD*=6.81), including one group of NC women (*N*=40) and one group taking OC (*N*=38), were tested twice over the course of one menstrual cycle. The NC women were tested during their follicular and luteal phases while the OC users were tested during the active phase of their OC. They completed an automated version of the Operation Span task (OSPAN; Unsworth et al., 2005). The OSPAN task involves completing simple math problems while simultaneously trying to remember a series of randomly generated letters.

**Results:**

There was no difference in WM capacity between NC women and OC users. However, we found a significant difference in the number of math errors (speed or accuracy) made by NC women. The number of math errors was higher (*M*=5.12) during the luteal phase than during the follicular phase (*M*=2.68). Moreover, we also found a significant difference in the number of math errors made by OC users (*M*=4.00) and NC women (*M*=2.68) in their follicular phase. The difference disappeared when the OC users were compared to NC women in their luteal phase.

**Conclusions:**

We found no difference in WM capacity between NC women and OC users. However, we found that NC women made more math errors during the luteal phase than during the follicular phase and that OC users made more errors than NC women in their follicular phase. The follicular phase has an increased level of estradiol whereas OC users experience a decrease in their levels of estradiol. Estradiol levels could be linked with math performance.

**Disclosure of Interest:**

None Declared

